# Hydrogen: a novel strategy for preventing and treating hearing loss

**DOI:** 10.4103/mgr.MEDGASRES-D-25-00274

**Published:** 2026-04-11

**Authors:** Ling Jin, Shiwang Tan, Ju Lai, Kai Fan, Shican Zhou, Yang Wang, Shaoqing Yu

**Affiliations:** Department of Otolaryngology, Tongji Hospital, School of Medicine, Tongji University, Shanghai, China

**Keywords:** age-related hearing loss, hearing loss, hydrogen, mechanism, noise-induced hearing loss, oxidative stress, prevention, progress, sensorineural hearing loss, treatment

## Abstract

**Facts**

1. Hydrogen selectively neutralizes harmful free radicals, protecting cochlear cells.
2. Animal studies confirm that hydrogen can reduce hearing damage caused by noise and drugs.
3. Clinical trials have shown that hydrogen inhalation can improve hearing in certain types of patients.
4. Hydrogen has strong diffusivity, allowing it to penetrate barriers and reach inner ear targets.

**Open Questions**

1. What is the most effective delivery method for hydrogen to reach the human cochlea?
2. Can hydrogen prevent or treat age-related hearing loss? Is experimental evidence still lacking?
3. Can the therapeutic effects observed in small-sample studies be replicated in large-scale trials?
4. Is long-term hydrogen therapy safe? What are its physiological impacts?
5. Beyond its antioxidant effects, what are the specific molecular targets of hydrogen in the auditory system?

Sensorineural hearing loss, often caused by irreversible damage to cochlear hair cells, is primarily mediated by oxidative stress triggered by aging, noise exposure, or ototoxic agents. Conventional treatments such as hearing aids or cochlear implants have limitations in accessibility and efficacy. Hydrogen (H_2_), a selective antioxidant with anti-inflammatory and anti-apoptotic properties, has demonstrated protective effects on various organ systems. This review evaluates the therapeutic potential of H_2_ in preventing and treating sensorineural hearing loss and aims to inform future research directions. In preclinical models of sensorineural hearing loss, H_2_ administration via inhalation, injection, or drinking water reduces reactive oxygen species accumulation, protects cochlear hair cells, and improves functional hearing markers (auditory brainstem response and distortion product otoacoustic emission). Clinical trials have shown that H_2_ inhalation improves hearing thresholds in patients with radiotherapy-induced hearing loss and idiopathic sudden sensorineural hearing loss. No serious side effects have been observed. H_2_’s high diffusion capacity and selective reactive oxygen species-scavenging properties support its role as a promising therapeutic for sensorineural hearing loss. While animal data are robust and initial clinical outcomes encouraging, further large-scale studies are warranted to validate efficacy, optimize delivery methods, and establish clinical protocols.

## Introduction

Hearing loss is the most common sensory impairment worldwide, affecting over 500 million people.^1^ Two to three out of every 1000 children have hearing loss in their first year of life,^2^ and the prevalence increases to 50% in adults over the age of 70 years.^3^ Older adults with oral speech-dependent and untreated acquired hearing loss are at an increased risk of developing neurodegenerative dementia.^4^,^5^,^6^,^7^ This increases the burden on individuals, families, and society. The current treatment for deafness is mainly symptomatic, that is, wearing hearing aids or cochlear implants, which many patients still find difficult to accept^8^; therefore, the prevention and treatment of hearing loss is a topic that needs more attention in otology.

Since 2007, a Japanese research team has been the first to discover the value of hydrogen (H_2_) in the treatment of cerebral ischemia-reperfusion injury,^9^ and ever since then, more research has been conducted on H_2_ as a therapeutic gas molecule, and a large number of studies have confirmed that H_2_ can be used to treat a variety of diseases, mainly in reducing ischemia-reperfusion injury in tissues and organs,^10^,^11^ reducing pulmonary hypertension,^12^ antitumor,^13^ improving body metabolism.^14^,^15^ and improving sepsis.^16^ However, few studies have been conducted in the field of otology. The main focus was on the application of H_2_ to hearing loss. In recent years, research has demonstrated that H_2_ can slow down or even reverse hearing loss caused by noise, ototoxic drugs, or aging in various experimental models. Therefore, exploring the mechanisms by which H_2_ is used for the prevention and treatment of hearing loss, as well as its potential clinical applications, can become a new direction in otological research.

Any structural or functional disorder in the transmitting, perceptive, or analytical integrative parts of the human auditory system can manifest as hearing loss of varying degrees and is collectively referred to as hearing loss in clinical practice. There are many ways to classify hearing loss, but the common ones are classified according to the nature and location of the lesion into two categories: organic hearing loss and functional hearing loss. Organic hearing loss can be classified into three types based on the location of the lesion: conductive hearing loss, sensorineural hearing loss (SNHL), and mixed hearing loss. SNHL is the most prevalent form of hearing impairment, including primarily noise-induced hearing loss (NIHL), age-related hearing loss (ARHL), and ototoxic drug-induced hearing loss, with increasing human lifespan, greater exposure to noise, and escalating probability of ototoxic medication usage.^1^,^3^,^17^ SNHL has progressively increased, significantly affecting individuals’ quality of life; and these causes involve oxidative stress, which has led to the investigation of novel antioxidant therapies, such as H_2_.^1^,^3^,^7^,^8^,^9^,^17^ H_2_’s unique combination of exceptional diffusive capacity and selective antioxidant properties renders it particularly noteworthy in the context of hearing loss when compared to conventional antioxidant approaches. Currently, numerous basic research studies have confirmed the beneficial effects of H_2_ on SNHL. Here, we present a systematic summary of the theoretical basis, mechanism, and progress of H_2_ research in this area to facilitate further research and the development of related clinical research in the future. Against the backdrop of global aging and the rising annual incidence of hearing loss, investigating safe and effective novel approaches for preventing and treating hearing loss holds profound significance. H_2_, in turn, offers a promising new direction for research into the prevention and treatment of hearing loss.

## Search Strategy

Relevant studies were systematically retrieved from PubMed, Scopus, and Web of Science using the keywords “hydrogen,” “hearing loss,” “sensorineural hearing loss,” and “noise-induced hearing loss.” Only literature from the past decade detailing cellular and molecular mechanisms, experimental interventions, or therapeutic outcomes was included.

## Mechanism of Hearing Loss

### Cytological mechanism: hair cell damage

**Figure 1:**
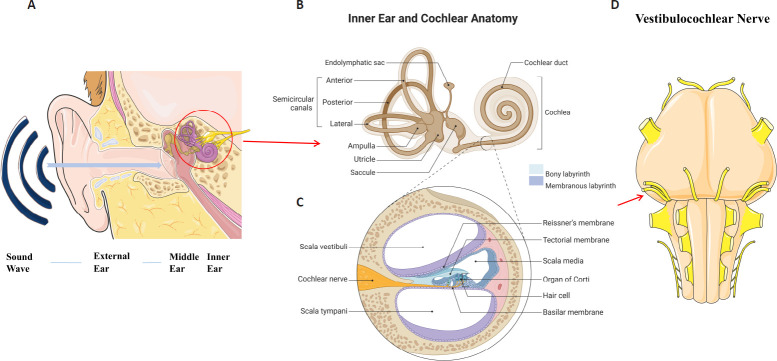
Normal auditory pathway. Sound causes vibrations in the ossicular chain (A), then the cochlea in the inner ear transmits vibrations of sound waves to the organ of Corti on the basilar membrane (B, C), this causes bioelectric changes in the inner and outer hair cells above, then releases chemical transmitters, and form the nerve impulses to the auditory center (D) and finally this becomes hearing in the cerebral cortex. Created with Tayasui Sketches.

In normal auditory physiology, acoustic energy is transmitted through the external auditory canal to the tympanic membrane, subsequently inducing mechanical vibrations in the ossicular chain (malleus, incus, and stapes) (**Figure 1A**). The cochlea in the inner ear transmits vibrations of sound waves to the organ of Corti on the basilar membrane (**Figure 1B** and **C**), which causes bioelectric changes in the inner and outer hair cells (OHCs) above, releases chemical transmitters, and forms nerve impulses to the auditory center (**Figure 1D**), and finally, hearing in the cerebral cortex.^18^ Information about sound is encoded by the cochlea and transmitted to the brain. When this neurotransmission is reduced or interrupted, hearing loss occurs. OHCs respond to vibrations and amplify them to increase shear along the basilar membrane and then stimulate the inner hair cells (IHCs). Loss of OHCs or a reduction in clinical activity can affect the activity of IHCs, resulting in hearing loss.^19^

Hair cells, the primary sensory cells in the auditory system of the inner ear, play a pivotal role in sound transduction. Damage to these cells constitutes a principal etiological factor in SNHL. Cellular signaling pathways underlying hair cell degeneration and their associated cell death mechanisms are intricately linked to the pathogenesis of SNHL.^20^ The degeneration of cochlear hair cells involves multiple cellular signaling pathways. Notably, the mitogen-activated protein kinase (MAPK) signaling pathway plays a pivotal role in mediating hair cell responses to diverse ototoxic stimuli.^21^ Exposure of the inner ear to ototoxic insults, such as noise trauma or ototoxic drugs, activates the MAPK signaling cascade, encompassing key subfamilies, including extracellular signal-regulated kinase, c-Jun N-terminal kinase, and p38 MAPK.^21^ Activated MAPK signaling modulates downstream transcription factors and effector molecules, thereby regulating critical cellular processes in cochlear hair cells, including survival, proliferation, and apoptosis.^21^ Furthermore, the phosphatidylinositol 3-kinase/protein kinase B (Akt) signaling pathway is involved in hair cell homeostasis.^22^ Under physiological conditions, this pathway promotes hair cell viability, whereas its dysregulation under pathological stress directly affects hair cell fate.^22^

Cochlear hair cell injury manifests through dysregulated activation of autophagic, apoptotic, and necrotic pathways. Moderate autophagy plays a cytoprotective role by maintaining intracellular homeostasis through selective clearance of damaged organelles (e.g., mitochondria) and attenuation of cytotoxic stress via the removal of toxic aggregates.^23^,^24^ However, under conditions of excessive pathological stimuli or dysfunctional autophagy, overactivation of autophagy may paradoxically precipitate hair cell demise.^23^,^24^ Prior work suggests that noise and ototoxic drugs dysregulate autophagy-lysosomal pathways, leading to the accumulation of damaged organelles in hair cells.^25^,^26^

Apoptosis is a common cell death pathway in hair cells.^27^ Multiple signaling pathways can induce hair cell apoptosis, including the mitochondrial and death receptor pathways.^27^ Cisplatin and noise exposure induce hair cell apoptosis via reactive oxygen species (ROS)-mediated activation of caspase-3 and mitochondrial depolarization.^28^,^29^

Under severe damage conditions, such as intense noise exposure or high-dose ototoxic drug exposure, necrosis of hair cells can occur, in which the excessive production of ROS plays a significant role.^20^ Acute noise trauma (> 130 dB SPL) mechanically disrupts hair cell stereocilia, triggering necrotic cell death.^26^

OHCs exhibit electromotile properties that are crucial for amplifying low-intensity acoustic signals, converting mechanical vibrations into electrical signals, and transmitting them to IHCs via synaptic connections with afferent neurons.^30^,^31^ IHCs constitute the principal site of auditory signal transduction and efficiently convert the amplified mechanoelectrical signals from OHCs into neural impulses, which are subsequently transmitted to the central auditory pathways via synaptic connections with afferent nerve fibers.^32^,^33^

Damage to both the OHCs and IHCs contributes to SNHL. The differential involvement of pathological mechanisms and cell death pathways mediates distinct patterns and severity of cellular damage in OHCs and IHCs, thereby generating heterogeneous SNHL phenotypes in terms of audiological characteristics and progression trajectories.^20^ Damage to the OHCs compromises the active amplification mechanism of auditory signals, resulting in diminished sensitivity to low-intensity sounds within the cochlea. Clinically, this pathophysiological alteration manifests as elevated hearing thresholds.^20^,^30^,^31^ OHCs act as cochlear amplifiers, enhancing basilar membrane vibrations through electromotility. Loss of these structures, whether induced by noise exposure or cisplatin administration, results in a significant decline in auditory function. This is evidenced by elevated auditory brainstem response (ABR) thresholds, indicating reduced hearing sensitivity and impaired distortion product otoacoustic emission amplitudes, reflecting compromised frequency selectivity.^34^,^35^ Conversely, damage to IHCs directly impairs the transmission of auditory signals to the central nervous system, resulting in an inability to accurately encode and relay sound information, which leads to hearing impairment.^20^,^32^,^33^ The IHCs are primary sensory transducers that convert mechanical stimuli into afferent neural signals via ribbon synapses with spiral ganglion neurons (SGNs). Although IHCs are more resistant to ototoxicity, synaptic loss (synaptopathy) between IHCs and SGNs disrupts auditory neurotransmission, contributing to “hidden hearing loss.”^36^

### Molecular mechanism: oxidative stress

There are several causes of SNHL, including aging, noise, ototoxic drugs, and infections. Ultimately, these causes ultimately lead to cochlear damage, leading to hearing loss.^20^ However, the specific location of cochlear damage varies.

ARHL or presbycusis is the most prevalent sensory impairment in older adults.^37^ It is characterized by a gradual decline in hearing function, primarily resulting from degeneration of the stria vascularis (SV), auditory hair cells, and SGNs.^38^ Damage to hair cells plays a critical role in the progression of ARHL. Oxidative stress has been identified as a major contributor to the onset and development of ARHL.^39^ Noise exposure causes the contraction of cochlear blood vessels and disrupts cell energy metabolism, which generates large amounts of free radicals, such as ROS,^40^ which can damage not only hair cells but also synapses of SGNs through glutamate excitotoxicity.^41^,^42^,^43^

Cisplatin and other ototoxic drugs can cause the death of cochlear hair cells and surrounding supporting cells.^44^ Infections (cytomegalovirus and other viruses) can cause inflammation of the lateral walls and vascular lines of the cochlea.^45^,^46^

**Figure 2:**
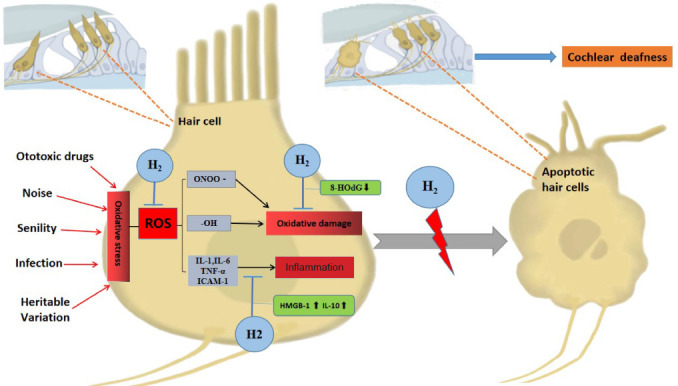
Mechanism of hydrogen on hearing loss. Hydrogen exerts protective effects via multiple pathways: it directly scavenges harmful ROS, inhibits oxidative damage, and modulates inflammatory responses. Ultimately, hydrogen blocks the process of hair cell apoptosis, thereby preventing cochlear deafness resulting from hair cell death. Created with Tayasui Sketches. ·OH: Hydroxyl radical; H_2_: hydrogen; HMGB-1: high mobility group box 1; ICAM-1: intercellular adhesion molecule 1; IL: interleukin; ONOO^−^: peroxynitrite; ROS: reactive oxygen species; TNF-α: tumor necrosis factor-α.

Oxidative stress drives the irreversible and progressive degeneration of cochlear hair cells.^47^ Oxidative stress causes the accumulation of hair cell peroxides, and these peroxides and other molecular reactions generate large amounts of free radicals in the cochlea. If these free radicals are not neutralized, they trigger apoptosis and necrosis of the hair cells, as well as damage the supporting cells surrounding the hair cells and disrupt the SGNs, among other effects, ultimately leading to hearing loss or tinnitus,^47^ as shown in **Figure 2**. Many studies have confirmed that excessive production of free radicals (e.g., ROS) is the main cause of SNHL.^48^,^49^,^50^ The primary intracellular site of ROS generation is the mitochondria. Oxidative stress-induced hearing loss is predominantly mediated by dysfunctional mitochondria.^51^ Excessive ROS can trigger progressive oxidative damage, resulting in mitochondrial dysfunction in cochlear hair cells. ROS not only diminishes mitochondrial membrane potential but also promotes calcium overload, further aggravating mitochondrial injury. This cascade ultimately leads to apoptosis or necrosis of hair cells, contributing to hearing loss.^52^ Another critical aspect is the impact of oxidative stress on cochlear blood flow and its bidirectional interplay. The cochlear vascular supply plays a pivotal role in maintaining the structural integrity and functional homeostasis of the auditory periphery. Oxidative stress can induce vascular disturbances within the cochlea, whereas ischemia-reperfusion injury conversely exacerbates ROS overproduction, creating a self-perpetuating pathogenic cycle.^51^,^53^ Given the exquisite sensitivity of cochlear microcirculation to hemodynamic alterations, even subtle perturbations in blood flow can initiate a pathophysiological cascade, culminating in detrimental effects on auditory transduction and hair cell viability.^53^

A series of diagnostic and therapeutic studies have been conducted.^54^,^55^,^56^,^57^,^58^,^59^,^60^,^61^,^62^,^63^ The use of an antioxidant stress mechanism to prevent deafness is undoubtedly feasible. Preventive and therapeutic strategies for hearing loss in preclinical studies primarily use antioxidants.^54^,^55^,^56^,^57^,^58^,^59^,^60^,^61^,^62^,^63^ Numerous studies have demonstrated that resveratrol in combination with N-acetylcysteine,^54^ eugenol,^55^ cranberry extract,^56^ ursolic acid,^57^ avocado oil extract,^58^ pomegranate peel extract,^59^ trehalose,^60^ curcumin,^61^ and allomelanin nanoparticles^62^ possess antioxidant properties capable of mitigating cochlear damage induced by ototoxic medications. Zheng et al.^63^ used the DNA methylation inhibitor RG108 to attenuate oxidative stress-induced DNA damage and apoptosis-mediated cell loss in the cochlea of mice after noise exposure to prevent noise-induced deafness.

## Potential Mechanism of Hydrogen on Hearing Loss

Despite extensive research on H_2_ in other fields, its application in otology remains underexplored. However, emerging evidence suggests its potential in protecting cochlear hair cells, warranting a focused review of current findings.

### Main mechanism: antioxidant stress

Currently, research on the preventive and therapeutic effects of H_2_ on hearing loss primarily revolves around its antioxidant mechanisms in counteracting cochlear damage. H_2_ is the smallest molecule in nature and is a colorless, tasteless, odorless, diatomic gas with reducibility properties. Ohsawa et al.^9^ were the first to discover that inhaling 2% H_2_ can selectively scavenge hydroxyl radicals (·OH) and peroxynitrite anions (ONOO-) from ROS and significantly improve oxidative stress injury caused by cerebral ischemia-reperfusion injury. H_2_ is considered to possess antioxidant stress injury properties. The molecular interactions between H_2_ and ROS involve two distinct mechanisms. First, H_2_ directly neutralizes cytotoxic ROS such as ·OH and ONOO-, through redox reactions, as demonstrated in multiple studies.^9^ This antioxidant property effectively mitigates oxidative damage to cellular components. Substantial evidence shows that molecular H_2_ exerts protective effects by significantly reducing DNA oxidation, as evidenced by decreased levels of 8-hydroxy-2’-deoxyguanosine, and inhibiting caspase-3 activation pathways in both SGNs and OHCs.^36^,^64^ Second, H_2_ regulates cellular signaling pathways. Notably, it modulates the nuclear factor erythroid 2-related factor 2 pathway, because it is the smallest gaseous molecule that facilitates membrane permeation and subsequent nuclear translocation. This facilitates nuclear factor erythroid 2-related factor 2 binding to antioxidant response elements, thereby upregulating the expression of cytoprotective enzymes.^65^ Many studies have found that H_2_ molecules can modulate the levels of oxidative stress markers and reduce the levels of malondialdehyde, 4-hydroxynonenal, 8-hydroxy-2’-deoxyguanosine, myeloperoxidase, and other lipid peroxidation products, while increasing the levels of superoxide dismutase, catalase, glutathione peroxidase, and other antioxidant enzyme activities.^48^,^66^,^67^,^68^

The overproduction of reactive ROS due to oxidative stress and necrotic apoptosis of cells is an important factor in the acute phase of cochlear damage. Many studies have found that the selective antioxidant, anti-damage, and anti-apoptotic properties of H_2_ also have positive effects on inner ear damage (**Figure 2**). Because H_2_ molecules are electrically neutral and much smaller than oxygen molecules, they can easily penetrate biological membranes and reach target sites such as mitochondria and nuclei that most antioxidants cannot effectively reach.^9^ This makes H_2_ easier than other antioxidants, such as N-acetylcysteine, eugenol, cranberry extract, to work inside damaged hair cells in the inner ear.^36^ Studies have found that H_2_ -rich water has a protective effect against SNHL in mice with mitochondrial dysfunction.^29^,^30^,^31^,^32^,^33^,^34^,^35^,^36^,^69^ A comparative analysis of H_2_ and currently employed antioxidants for hearing loss management is summarized in **Table 1**.^25^,^26^,^28^,^29^,^34^,^35^,^36^,^52^,^55^,^56^,^57^,^58^,^59^,^60^,^64^,^69^,^70^,^71^

**Table 1:** Comparison of hydrogen and other antioxidants for hearing loss

Drug	Experimental model	Administration	Mechanism	Type of hearing loss	Reference
Hydrogen	Rodents (gerbil/guinea pig/mouse)	Inhalation; IP/IV injection; oral	Potent antioxidant (ROS scavenging, lipid peroxidation reduction); anti-inflammatory; protects hair cells/synapses	NIHL; ODIHL ischemia-induced; temporary threshold shift; AN	25 26 28 29 34 35 36 64 70 71
Pomegranate peel extract	BALB/c mice (D-galactose-induced aging)	Subcutaneous D-gal (800 mg/kg/d) + oral pomegranate peel extract (17/34 mg/kg/d)	Modulates PNUTS/PP1 pathway, inhibits oxidative stress (↓4-HNE, p53, caspase-3; ↑MDM2)	ARHL	59
Avocado oil extract (DKB122)	HEI-OC1 cells (*in vitro*)	*In vitro* treatment (25 μg/mL)	Enhances glutathione metabolism genes (Hmox4, Gsta4), inhibits ROS & cytokines (TNF-α, IL-6), activates autophagy (↑LC3-II, ↓p62)	ODIHL; neomycin-induced ototoxicity	58
SIRT3 inhibitor (3-TYP)	C57BL/6 mouse cochlear organ culture (*in vitro*)	H_2_O_2_ (0.5–1 mM) + 3-TYP (50 μM) in culture medium	SIRT3 deficiency exacerbates mitochondrial damage (↓MMP, ↑ROS), inhibits FOXO3A/SOD2 pathway, promotes hair cell apoptosis	SNHL; ROS-induced cochlear injury	52
Eugenol	Female Sprague–Dawley rats	Intraperitoneal injection (100 mg/kg/d for 5 d)	Enhances SOD/GPx activity, reduces MDA/8-OHdG, improves cochlear morphology	ODIHL; cisplatin-induced ototoxicity	55
Bilberry extract	Male Wistar rats	Intraperitoneal injection (100/200 mg/kg for 8 d)	Reduces TOS/OSI, alleviates cochlear degeneration (dose-dependent)	ODIHL; cisplatin-induced ototoxicity	56
Trehalose	C57BL/6J mouse cochlear culture/HEI-OC1 cells	*In vitro* treatment (10 mM)	Activates TFEB-mediated autophagy (↑LC3-II), inhibits apoptosis (↓cleaved caspase-3), improves mitochondrial function	ODIHL; cisplatin-induced ototoxicity	60
Ursolic acid	BALB/c mice	Intraperitoneal injection (80 mg/kg/d for 5 d)	Suppresses TRPV1/Ca²^+^/calpain pathway, reduces 4-HNE production and caspase-3 activation, synergizes with cisplatin’s anticancer effects	ODIHL; cisplatin-induced ototoxicity	57
Taurine, coenzyme Q10, hydrogen water	CBA/J mice (Germanium dioxide-induced mitochondrial dysfunction)	Oral intake via drinking water for 3 months (Taurine 0.3%, CoQ10 150 µM, H_2_ > 0.4 mM)	Scavenges ROS; prevents downregulation of mitochondrial respiratory chain genes (e.g., *Atp5a1*, *Cox5a*); inhibits apoptosis and degeneration of stria vascularis and spiral ganglion neurons	SNHL; mitochondrial dysfunction-associated hearing loss	69

4-HNE: 4-Hydroxynonenal (or 4-hydroxy-2-nonenal); 8-OHdG: 8-hydroxy-deoxyguanosine; AN: auditory neuropathy; ARHL: age-related hearing loss; FOXO3A: Forkhead box O3A; GPx: glutathione peroxidase; IP: intraperitoneal; IV: intravenous; LC3-II: microtubule-associated protein 1 light chain 3-II; MDA: malondialdehyde; MDM2: murine double minute 2; MMP: mitochondrial membrane potential; NIHL: noise-induced hearing loss; ODIHL: ototoxic drug-induced hearing loss; OSI: oxidative stress index; PNUTS: protein phosphatase 1 nuclear targeting subunit; PP1: protein phosphatase 1; ROS: reactive oxygen species; SIRT3: Sirtuin 3; SOD: superoxide dismutase; TFEB: transcription factor EB; TOS: total oxidant status; TRPV1: transient receptor potential vanilloid 1.

### Other mechanisms

#### Immunomodulation

In hearing loss, inflammation is often caused by the activation of immune cells and the subsequent release of inflammatory factors. H_2_ can modulate the immune system and reduce excessive inflammation. It works by inhibiting the nuclear factor kappa-B signaling pathway, reducing the secretion of inflammatory factors, such as interleukin (IL)-6 and tumor necrosis factor-α, and suppressing the activation of T and B cells, thereby reducing inner ear inflammation.^26^

#### Mitochondrial protection

Mitochondria are the energy centers of cells and their dysfunction is closely linked to various pathological conditions. H_2_ enhances mitochondrial function and mitigates cellular damage caused by hypoxia or energy deficiency. By reducing ROS accumulation within the mitochondria, H_2_ protects cochlear cells from the adverse effects of metabolic disorders, thereby delaying the onset of hearing loss.69 H_2_ protects OHCs by preserving mitochondrial function and reducing lipid peroxidation.^29^,^36^

One important mechanism of hearing loss is the death of cochlear hair cells, particularly via apoptotic pathways. H_2_ regulates the expression of apoptosis-related genes and inhibits programmed cell death. In NIHL, H_2_ reduces the death rate of cochlear hair cells by inhibiting the activity of pro-apoptotic factors, such as p53 and Bax.^25^ H_2_ inhalation mitigates synaptophysin depletion in IHCs and preserves synaptic integrity.^36^ H_2_’s dual protection of hair cells and synapses (via antioxidant and anti-apoptotic effects) explains its efficacy in diverse SNHL models.^28^,^70^

## Experimental Research on Hydrogen for Hearing Loss

In recent years, basic research on H_2_ in the prevention and treatment of hearing loss is still relatively weak, and research has mainly focused on NIHL and ototoxic drug-induced hearing loss, both of which fall under SNHL. The conclusions of these studies are shown in **Table 2**.^25^,^26^,^28^,^29^,^34^,^35^,^64^,^70^,^71^,^72^,^73^,^74^ ARHL, a subtype of SNHL, theoretically suggests that molecular H_2_ may mitigate cochlear hair cell senescence and degeneration through conserved antioxidant mechanisms similar to its established otoprotective effects. However, there is a critical knowledge gap in the literature due to the absence of dedicated investigations validating this hypothesis, which is an important research direction for developing targeted auditory neuroscience.

**Table 2:** Basic research on hydrogen in the prevention and treatment of hearing loss

Study	Year	Experimental animal	Group	Cause of hearing loss	Intervention	Evaluation index	Result
Lin et al.^35^	2011	Male guinea pigs	Two groups (17/group): ▪Normal water treated ▪H_2_ water treated	3-h noise (115 dB SPL, 4 kHz octave band)	Drinking H_2_-rich water	ABR; DPOAE	ABR: H_2_ group showed significantly better thresholds at 2/4 kHz on days 1, 3, and 14 post-noise DPOAE: H_2_ group exhibited higher I/O curve amplitude during recovery
Zhou et al.^25^	2012	Guinea pigs	Four groups (15/group): ▪HS + noise (i.p. HRS) ▪NS + noise (i.p. NS) ▪Noise alone ▪No treatment	4-h noise (115 dB SPL, 4000 ± 100 Hz)	Twice a day intraperitoneally	ABR; DPOAE; Histological analysis of the cochlea	ABR: HS + noise group had significantly lower thresholds DPOAE: HS + noise group exhibited higher amplitudes Cochlear histology: Noise alone & NS + Noise groups showed significantly more missing OHCs *vs*. HS + noise & untreated groups
Qu et al.^64^	2012	Male Mongolian gerbils	Four groups (6/group): ▪Saline ▪Saline + H_2_ ▪Ouabain ▪Ouabain + H_2_	Ouabain-induced AN model	H_2_ inhalation for 60 min	ABR; DPOAE; Histological assessment; TUNEL staining; Immunofluorescent staining for activated caspase-3; Caspase-3 activity	ABR: H_2_ (2/4%) reduced click/tone-evoked threshold shifts at 4/8/16 kHz exposed animals. DPOAE: No amplitude changes at 4/8/16 kHz in ouabain or H_2_ groups. Cochlear morphology: H_2_ (2%) preserved SGN density (all cochlear turns) *vs*. ouabain controls; H_2_ ↓ ouabain-induced SGN apoptosis (*P* < 0.01); No HC morphological changes observed
Kikkawa et al.^28^	2014	ICR mice (Japan SLC)	20 cochlear explants	Medium containing cisplatin	H_2_ was dissolved directly into the media	Cell survival assay (IHCs and OHCs); Detection of ROS by fluorescent indicators	Cell survival assay (IHCs and OHCs): Whereas the addition of H_2_ significantly increased the numbers of remaining auditory hair cells. Detection of ROS by fluorescent indicators: Formation of hydroxyl radicals was successfully reduced in hydrogen-treated cochleae
Kurioka et al.^34^	2014	Female Hartley guinea pigs	Five groups (6/group): ▪Normal ▪non-treated ▪0.5%, 1.0%, 1.5% H_2_-treated	One octave band noise centered at 4 kHz at 121 dB SPL for 5 h	Inhaled hydrogen gas	ABR; Quantitative assessment of hair cell loss; 8-OHdG	ABR: 1.0/1.5% H_2_ groups showed ↓ threshold shifts *vs*. untreated (*P* < 0.05). OHCs survival: Higher OHCs survival in H_2_ groups (basal turn, *P* < 0.01 *vs*. control). 8-OHdG: H_2_ groups exhibited ↓
Chen et al.^29^	2014	Guinea pigs	Three groups (*n* = 55): ▪HRS (*n* = 20) ▪NS (*n* = 20) ▪Control (*n* = 15)	Narrow band noise (2.5–3.5 kHz 130 dB SPL, 1h)	Intraperitoneal injection	ABR; DPOAE; Free radicals in the cochlea (LPO, MDA, and ·OH)	HRS group had less of an ABR threshold shift; DPOAE amplitude: HRS group significantly higher; Free radical detection: LPO, MDA, and ·OH levels were significantly lower in the HRS group
Chen et al.^26^	2017	Guinea pigs	Three groups (6/group): ▪HRS ▪NS ▪Control	Narrow band noise centered at 2.5–3.5 kHz, a level of 130 dB SPL for 1 h	Intraperitoneal injection	ABR; 8-HOdG; IL-1, IL-6, IL-10, TNF-α, ICAM-1, HMGB-1	HRS group had less ABR threshold shift; 8-HOdG: Significantly decreased in the HRS group; IL-1, IL-6, TNF-a, and ICAM-1: HRS group were dramatically reduced; HMGB-1 and IL-10: HRS group were significantly higher
Fransson et al.^70^	2017	Albino guinea pigs	Four groups (*n* = 34): ▪Cispt (*n* = 11) ▪Cispt + H_2_ (*n* = 11) ▪H_2_ (*n* = 5) ▪Control (*n* = 7)	Single intravenous injection of cisplatin	Immediately followed by 60-min administration of gaseous H_2_	ABR; Hair cell loss; IHCs findings in cisplatin-damaged subcells	ABR: No threshold shifts in H_2_ *vs*. Control; HC loss: Cispt + H_2_ preserved more OHCs and IHCs *vs*. Cispt alone; Molecular targets: Cispt↓OCT2 (pillar cells, stria vascularis) & CTR1 (IHCs synapses/Deiters/stria); H_2_ prevented ≥ 80% of Cispt-induced OCT2/CTR1 reductions
Ogawa et al.^71^	2018	Adult male Mongolian gerbils	Four groups (6/group): ▪Non-ischemia/saline ▪Non-ischemia/HRS ▪Ischemia/saline ▪Ischemia/HRS	Transient cochlear ischemia (15-min bilateral vertebral artery occlusion)	Intravenously administration	ABR; IHCs	ABR: The increase in ABR threshold was 11.7 ± 2.6 dB at 8 kHz; IHCs: IHCs loss was 7.5 ± 2.1% at the basal turn on day 7
Fransson et al.^36^	2021	Guinea pigs (both sexes)	Six groups (8/group): ▪Acute + H_2_ ▪Acute + Air ▪1w + H_2_ ▪1w + Air ▪2w + H_2_ ▪2w + Air	Broadband noise (2–20 kHz) for 2 h at 115 ± 2 dB SPL)	Over 1 h through a facial mask	ABR; Hair cell loss; Changes to the IHCs and OHCs synapse; Region and immune responses (Synaptophysin and the distribution of Iba1)	ABR: H_2_ -treated group showed ↓ thresholds *vs*. control at post-exposure W2; OHCs survival: H_2_ preserved OHCs across cochlear turns; Synapse/inflammation: ↑ SYN^+^ puncta in IHCs (H_2_ *vs*. control); H_2_ induced ↑ Iba1 in stria vascularis (SV); Repeated H_2_ administration enhanced therapeutic efficacy.
Videhult Pierre et al.^72^	2023	Guinea pigs	Three groups (*n* = 26): ▪Noise group (*n* = 11) ▪Noise + H_2_ group (*n* = 10) ▪H_2_ group (*n* = 5)	Exposed to 400 short sound impulses (~156 dB SPL) for approximately 3 min	H_2_ inhalation (2mol%; 500 mL/min) for 1 h	ABR; Quantification of hair cells	ABR: Noise↑ thresholds (*vs*. 20 dB SPL in Noise + H_2_ group); OHCs loss: Noise: 8.8% (apical)/53% (mid)/14% (basal); Noise + H_2_: 3.5%/22%/1.2% (basal *P* = 0.003 *vs*. Noise); IHCs loss: Noise: 0.1% (apical)/14% (mid)/3.5% (basal) Noise + H_2_: 0%/2.8%/0% (apical *P* = 0.033, basal *P* = 0.048)

8-HOdG: 8-Hydroxy-desoxyguanosine; ABR: auditory brainstem response; AN: auditory neuropathy; DPOAE: distortion product otoacoustic emission; H_2_ : hydrogen; HMGB1: high mobility group box-1 protein; HRS: hydrogen-rich saline; HS: hydrogen saline; ICAM-1: intercellular cell adhesion molecule-1; IHCs: inner hair cells; IL: interleukin; LPO: lipid peroxidation; MDA: malondialdehyde; NIHL: noise-induced hearing loss; NS: normal saline; OHCs: outer hair cells; ROS: reactive oxygen species; SGN: spiral ganglion neuron; TNF-α: tumor necrosis factor-α.

### Prevention

NIHL is a leading cause of acquired SNHL and is characterized by the loss of cochlear hair cells, synapses, and nerve endings. It is strongly associated with excessive production of reactive ROS in the cochlea.^73^ Extensive evidence suggests that oxidative stress and mitochondrial dysfunction triggered by high-intensity noise exposure are key mechanisms underlying the development of NIHL.^29^,^48^,^66^,^74^,^75^,^76^ Currently, there are no effective pharmacological treatments for NIHL, primarily because of the limitations imposed by the blood-labyrinth barrier on drug delivery to the inner ear. H_2_, with its strong diffusion properties, offers an advantage in this regard as it can easily reach cochlear hair cells and damaged mitochondrial targets in the inner ear, exerting selective antioxidant effects.

Research on H_2_ in hearing protection has revealed its multifaceted mechanisms. In the NIHL, existing studies primarily focus on oxidative stress mitigation, as exemplified by a pivotal animal study.^29^ The experimental model demonstrated that H_2_-saturated saline administration preserved auditory function and cochlear morphology through three key mechanisms: (1) reducing lipid peroxidation, (2) maintaining mitochondrial integrity, and (3) preventing hair cell structural damage. This foundational work, among other preclinical studies^25^,^26^,^34^,^35^,^70^ analyzed in our review, collectively supports H_2_’s antioxidant capacity in NIHL prevention, though the precise molecular pathways warrant further investigation. Another study in 2017 explored the molecular mechanism of the protective effect of H_2_-saturated saline on NIHL.^26^ The cochlear concentrations of oxidative stress markers, particularly 8-hydroxy-2′-deoxyguanosine, and proinflammatory mediators, including IL-1, IL-6, tumor necrosis factor-α, and intercellular adhesion molecule-1, were significantly decreased in guinea pigs treated with H_2_-saturated saline compared to those in the normal saline control group. Conversely, the expression levels of high mobility group box 1 and the anti-inflammatory cytokine IL-10 were markedly elevated in the H_2_-saturated saline group relative to those in the normal saline controls, suggesting that H_2_ may inhibit noise-induced cochlear inflammation by inhibiting the expression of proinflammatory cytokines (IL-1, IL-6, tumor necrosis factor-α, and intercellular adhesion molecule-1) and inducing the expression of anti-inflammatory cytokines (high mobility group box 1, and IL-10).^26^ This study revealed for the first time that the protective effects of H_2_-saturated saline on NIHL are related to both antioxidant and anti-inflammatory activities.^26^

Kurioka et al.^34^ compared relevant indicators after inhalation of H_2_ in guinea pigs exposed to noise and confirmed that H_2_ could prevent NIHL by reducing ROS in the inner ear and that H_2_ administration during the acute phase of noise damage could reduce hair cell damage and loss. Lin et al.^35^ exposed guinea pigs to 115 dB SPL 4-kHz octave noise for 3 hours before receiving plain or H_2_-rich water for 14 days. H_2_-treated guinea pigs exhibited a greater amplitude of distortion product otoacoustic emission input/output growth function magnitude during recovery, which suggests that H_2_ promotes hair cell recovery and attenuates noise-induced temporary hearing loss. Zhou et al.^25^ suggested that H_2_-rich saline could reduce experimental NIHL in guinea pigs, partially by preventing death of cochlear hair cells after intense noise exposure. Ogawa et al.^70^ investigated the protective effect of H_2_-rich water on ischemic injury in the gerbil cochleae and concluded that it is an effective antioxidant. Intravenous administration of H_2_-rich saline effectively prevented acute hearing loss caused by temporary cochlear ischemia.

### Treatment

Basic research on the therapeutic effects of H_2_ on hearing loss is still primarily focused on NIHL, likely due to the relatively well-established animal models associated with this condition. Fransson et al.^36^ found that the protective effect on hair cells by studying the inhalation of H_2_ in guinea pigs after exposure to noise, and therefore concluded that H_2_ is a therapeutic treatment for noise-induced deafness. In 2023, their team published another preclinical in vivo study.^72^ The results showed noise + H_2_ group inhaled 2 mol% H_2_ for 1 hour daily after noise exposure. Using ABR and hair cell loss as evaluation indicators, the results showed that the ABR threshold in the noise + H_2_ group was lower than that in the noise group after noise exposure, and the loss rate of IHCs and OHCs was also lower in the noise + H_2_ group than in the noise group. Therefore, it is believed that timely inhalation of H_2_ after exposure to noise in experimental animals could alleviate noise-induced acoustic injury.^72^ These two studies only presented observations without analyzing or discussing the underlying mechanisms, but they also suggested that the effect was mainly related to the antioxidant and anti-inflammatory activities of H_2_.^36^,^72^

Qu et al.^64^ found that H_2_ reduced apoptosis in auditory neuropathy through a model study of auditory neuropathy in gerbils using H_2_ and concluded that H_2_ may be a potential drug for the treatment of auditory neuropathy, as gas diffusion provides an effective inner ear treatment, and that H_2_ is a commonly used gas for acute traumatic cochlear disease.^77^ Based on the non-targeted metabolomics protocol of the perilymphatic duct with HILIC-UHPLC-Q-TOF-MS, some researchers found that there was a significant difference in the metabolome of lymphatics between guinea pigs exposed to noise with and without H_2_ treatment.^78^ A study confirmed that H_2_ could alleviate cochlear oxidative stress damage caused by noise.^79^

Aminoglycoside antibiotics and cisplatin are ototoxic drugs that can cause SNHL, which is closely related to oxidative stress and acidification of the inner ear microenvironment.^80^ In basic research on H_2_ for treating hearing loss, studies have not only focused on NIHL but also investigated the therapeutic effects of H_2_ on cisplatin-induced hearing loss. In 2012, an animal study found that inhalation of H_2_ reduced cisplatin-induced ototoxicity by decreasing oxidative stress.^79^ Kikkawa et al.^28^ showed that H_2_ molecules could reduce the damage caused by cisplatin in cochlear auditory hair cells. They studied the effect of culturing mouse cochlear explants in media containing different concentrations of cisplatin on H_2_ dissolved in the media, and found that adding H_2_ significantly increased the number of remaining auditory hair cells. Hydroxyphenyl fluorescein staining of spiral ganglia showed that it reduced the formation of hydroxyl radicals in the H_2_-treated cochlea, suggesting that H_2_ protects cochlear hair cells of the auditory system from cisplatin-induced free radicals. Similarly, Fransson et al.^71^ studied cisplatin-induced ototoxicity (permanent deafness and tinnitus) and found that H_2_ inhalation reduced cisplatin-induced ototoxicity at the functional, cellular, and subcellular levels after inhalation of H_2_ in intravenously cisplatin-injected mice.

## Clinical Research of Hydrogen on Hearing Loss

Compared with animal studies, there are even fewer clinical studies on the use of H_2_ for treating deafness. However, some scholars have made significant progress in this field. Kong et al.^81^ reported a prospective study on H_2_ inhalation for the treatment of hearing loss in patients with nasopharyngeal carcinoma after radiotherapy. They observed 17 patients with nasopharyngeal carcinoma after radiotherapy, with inhalation of H_2_ for 3–6 hours per day. The inhaled H_2_ flow rate was 3 L/min, with an H_2_ concentration of 67% (and oxygen content of 33%).^81^ Seventeen patients (34 ears) were divided into radiotherapy-only group (22 ears) and concurrent chemoradiotherapy group (12 ears). After 4 weeks of H_2_ inhalation, 17 patients showed improved Eustachian tube dysfunction score, decreased air-conduction thresholds and bone-conduction thresholds. Both groups exhibited progressive threshold reductions across 12 weeks, with the lowest values observed at 12 weeks. No adverse reactions, such as nasal bleeding, occurred during H_2_ inhalation, and it was found that H_2_ inhalation can improve the hearing of patients who undergo long-term radiotherapy and obtain a more significant curative effect with the prolongation of treatment time.^81^ This study found that inhaling H_2_ was effective in mitigating hearing loss, likely due to H_2_’s ability to regulate oxidative stress and inflammation, protect cells, and improve blood perfusion. However, the sample size of this study was small, with only 17 cases, and further research is required to confirm these findings.

Okada et al.^82^ in Japan treated and analyzed 65 patients with idiopathic sudden sensorineural hearing loss (ISSNHL) from 2019 to 2022. In the treatment group (*n* = 31), H_2_ inhalation therapy was administered along with conventional hormone therapy and other drugs. The control group received the same pharmacological regimen as the ambient air inhalation group (*n* = 34). Three months later, there was no significant difference between the groups in absolute thresholds. However, the H_2_ group demonstrated superior threshold improvement compared to controls. No H_2_ -associated adverse events were documented, and inhalation of H_2_ significantly improved therapeutic outcomes in patients with diabetes and severe hearing loss.^82^ These results suggest that H_2_ therapy may be effective for ISSNHL treatment and that H_2_ is expected to be used in various types of hearing loss treatments in the future. Despite the promising outcomes reported by Okada et al.,^82^ a search of major international registries such as ClinicalTrials.gov reveals a paucity of registered clinical trials focusing on hydrogen therapy for hearing loss. This absence indicates that clinical translation is still in its nascent stages and highlights a critical gap in the field.

## Limitations and Future Direction

H_2_ is considered a safe medical gas molecule. Extensive research and practice have shown that even with long-term, high-dose use, it generally does not cause significant harm to the human body.^83^ Currently, there are no clear contraindications to H_2_ therapy. Due to the challenges in continuous H_2_ delivery, such as H_2_ leakage, H_2_ embrittlement of metal, chemical compatibility (reduced elasticity of encapsulation materials), and conflicts between cost and utilization efficiency, current research tends to focus on the development and application of compact H_2_ generators.

Currently, hydrogen therapy for SNHL has not yet received formal approval from major regulatory agencies such as the U.S. Food and Drug Administration. While hydrogen is generally recognized as safe for consumption as a food additive in the United States, its clinical application as a therapeutic medical gas for hearing impairment remains in the investigational stage.

A limitation of this review is the relatively small number of relevant studies, particularly clinical research, which may introduce a certain degree of bias. It is anticipated that with the accumulation of more related literature in the future, a systematic, comprehensive summary covering both mechanisms and clinical applications can be achieved.

## Conclusion and Perspective

Various animal studies have suggested that H_2_, as an antioxidant, has protective and therapeutic effects against damage to hair cells in the cochlea. The small molecular weight of H_2_ has a strong diffusion function, which can rapidly reach the hair cells of the cochlea and selectively neutralize harmful ROS, thus reducing swelling and apoptosis of hair cells caused by ROS and thereby preventing and treating SNHL.

There are only two clinical studies on H_2_ therapy for deafness so far, so more studies should be performed. Addressing translational barriers from animal models to human applications would be scientifically valuable, including systematic investigations into H_2_’s bioavailability, rationalized administration routes (e.g., inhalation *vs*. nanocarrier delivery), and optimization of administration timing, modalities, and dosage regimens. This foundation will enable the transition to large-scale clinical trials evaluating combinatorial therapeutic approaches with existing interventions along with longitudinal studies assessing the safety of chronic H_2_ exposure.

**Figure 3:**
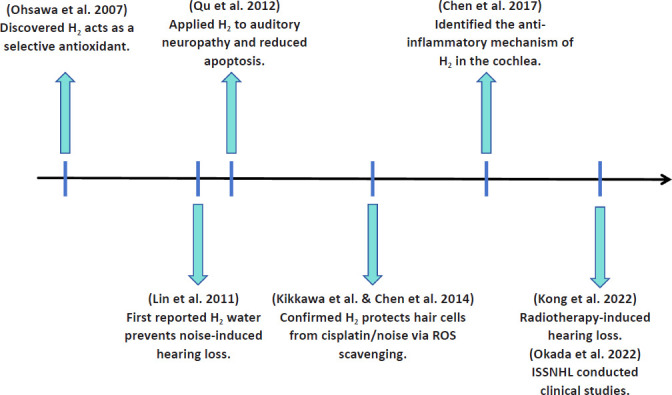
The key milestones in the development of hydrogen therapy for hearing loss. This timeline illustrates the evolution of hydrogen medicine in otology. Created with WPS Office (version: 12.1.0.24034). H_2_: Hydrogen; ISSNHL: idiopathic sudden sensorineural hearing loss; ROS: reactive oxygen species.

Although the current evidence remains preliminary, these efforts hold promise for bridging the translational gap between preclinical promise and clinical implementation (**Figure 3**). With sustained multidisciplinary collaboration, molecular H_2_ may emerge as a safe and effective novel strategy for preventing and treating SNHL across diverse etiologies.

## Data Availability

*No additional data are available.*
